# Cerebellar volume alterations are associated with cognitive dysfunction and fatigue in patients with systemic lupus erythematosus

**DOI:** 10.1186/s41927-026-00671-7

**Published:** 2026-07-02

**Authors:** Zahra Makdad Najeeb, Grégory Kuchcinski, Kristoffer Alexander Zervides, Jimmy Lätt, Tim Salomonsson, Petra Nilsson, Anders Bengtsson, Laura E. M. Wisse, Pia C. Sundgren, Andreas Jönsen

**Affiliations:** 1 Section of Diagnostic Radiology, Department of Clinical Sciences Lund, Lund University, Lund, Sweden; 2 Univ. Lille, CHU Lille, U1172 - LilNCog - Lille Neuroscience & Cognition, Inserm, Lille, F-59000 France; 3 Univ. Lille, CNRS, Inserm, CHU Lille, Institut Pasteur de Lille, 41 - UAR 2014 - PLBS, Lille, F-59000 France; 4 Department of Clinical Sciences Lund, Rheumatology, Lund University, Skåne University Hospital, Lund, Sweden; 5 Department of Medical Imaging and Physiology, Skåne University Hospital, Lund, Sweden; 6 Department of Clinical Sciences Lund, Neurology, Lund University, Skåne University Hospital, Lund, Sweden; 7 Lund Bioimaging Centre, Lund University, Lund, Sweden

**Keywords:** Systemic lupus erythematous (SLE), Cerebellum, Cerebellar lobules, MRI

## Abstract

**Background:**

Systemic lupus erythematosus (SLE) is a systemic autoimmune disease targeting multiple organ systems, including the nervous system. The cerebellum may be involved in prevalent neuropsychiatric manifestations, such as depression and cognitive dysfunction, alongside other prevalent SLE manifestations such as pain and fatigue. We aim to compare cerebellar volumes in SLE patients and healthy individuals (HI) and assess the correlation between cerebellar volumes and cognitive impairment, fatigue, pain, and depression in SLE.

**Methods:**

72 female SLE patients and 25 age- and sex-matched HI underwent 3 tesla magnetic resonance imaging (MRI), clinical evaluations, and cognitive testing. T1-weighted MRI scans were segmented using CEREbellum Segmentation (CERES) volBrain automatic segmentation. Extracted cerebellar lobule volumes (normalized to total cerebellar volume) were compared between SLE and HI using analyses of covariance (ANCOVA). In regions showing significant volume changes between SLE and HI, the relationship between volume and clinical scores for cognitive impairment, fatigue, pain, and depression was analyzed using either ANCOVAs or partial correlation analyses.

**Results:**

Lobular analysis of the cerebellum revealed significant (*p* < 0.05) region-specific volume alterations in SLE compared with HI. Bilateral and left lobule IV volumes were larger, while bilateral, right, and left lobule VIIB volumes were smaller in SLE compared to HI. Smaller white matter volumes in right lobule VIIIA, and right lobule X and larger white matter volumes in lobule crus I were observed in SLE compared to HI. In SLE, smaller VIIB volumes were significantly correlated with poorer cognitive performance (both complex attention and cognitive flexibility), and with higher fatigue scores.

**Conclusion:**

The findings suggest that the cerebellum, and particularly lobule VIIB, is involved in SLE and may contribute to both cognitive dysfunction and fatigue in patients with systemic lupus erythematosus.

**Trial registration:**

Not applicable.

**Supplementary Information:**

The online version contains supplementary material available at 10.1186/s41927-026-00671-7.

## Background

Systemic lupus erythematosus (SLE) is a chronic systemic autoimmune disease with multiorgan involvement, including the brain [1]. Common symptoms associated with SLE include cognitive dysfunction, depression, pain, and fatigue, resulting in a reduced ability to work and a poor health-related quality of life [2–6]. A patient-reported survey shows that fatigue and pain are two of the most common symptoms in SLE, and, alongside anxiety and depression, among the most burdensome for patients [7].

Patients with SLE that present with neuropsychiatric (NP) manifestations are often classified as neuropsychiatric systemic lupus erythematosus (NPSLE), which is defined by the American College of Rheumatology (ACR) as 19 neurological and psychiatric manifestations, including depression and cognitive dysfunction, arising from pathology in the central and peripheral nervous system where other potential causes have been ruled out [8]. The ACR NP case definitions have been criticized for being too inclusive, and a more restrictive classification model of NPSLE has been employed by the Systemic Lupus International Collaborating Centers (SLICC) group [8–11].

NP manifestations may precede the diagnosis of SLE and can occur at any point throughout the course of the disease [8]. One common NP manifestation in SLE is cognitive dysfunction, with an estimated prevalence of 38%. Common affected cognitive domains are memory, attention, psychomotor speed and verbal fluency [12]. Among these, memory, attention and psychomotor speed have been consistently related to structural brain abnormalities in SLE [13]. Depression as an NP manifestation of SLE has high variability, with an estimated prevalence of depressive symptoms of 35.2% [14]. Studies have found structural and functional cerebellar alterations, including grey matter volume loss and white matter fiber tract damage, in brain imaging of patients with depression [15], albeit not specifically in the cerebellum of patients with SLE.

Pain and fatigue, although not considered in the ACR-NP case definitions, are two closely associated central symptoms in SLE. Patients with SLE with NP involvement often tend to present with more severe fatigue levels compared to patients with SLE with no NP manifestations [5, 16]. Neuroimaging studies in patients with SLE with fatigue have shown various results regarding the correlation between fatigue and cerebral damage, especially focusing on white matter microstructure and white matter lesions [5, 17–21].

Given the substantial burden of pain, and fatigue in SLE, and the presence of structural brain abnormalities found in patients with SLE, there is a need to better understand how these symptoms relate to structural brain alterations. The cerebrum is a well-researched region in SLE, but the cerebellum is a region far less studied. The cerebellum is a brain region typically known for its motor roles, such as maintaining balance, coordination, muscle tone adjustments, and motor control [22], but is increasingly recognized for its role in cognition, fatigue, and pain [23–27].

The cerebellum is functionally divided into three: the “sensorimotor cerebellum”, consisting of lobules I-VI and lobule VIII, the “cognitive cerebellum”, consisting of the vermis and lobules VI, VIIA (including crus I and crus II) and VIIB, and the “vestibular cerebellum”, consisting of lobules V-VII and IX-X [23]. Cerebellar functions are highly compartmentalized, with specific cerebro-cerebellar pathways connecting different cerebellar regions to the cerebral cortex, in line with the cerebellum containing around 80% of all neuronal cells [23, 28]. Pathology in the cerebellum is often associated with ataxia, dysmetria, dysarthria, and oculomotor abnormalities [24], but its role has also been implicated in cognition. An example of the cerebellum’s implication in cognition is the cerebellar cognitive affective syndrome, which is characterized by impaired executive functions, visual-spatial processing, personality changes, language, verbal and visual learning impairment [23–25] and has been associated with damage to lobule VI, VII, and possibly IX, all of which belong to the posterior cerebellum [24].

While neurocognitive deficits are linked to cerebellar alterations in several inflammatory and ischemic disorders, cerebellar involvement in SLE remains sparsely investigated [29–31]. Cerebellar manifestations, including nystagmus, ataxia, tremor, and fine motor skill impairment, have been related to cerebellar volume loss in previous case reports of patients with SLE [31–38]. In the literature, cerebellar involvement in SLE in the form of cerebellar atrophy has been primarily reported in case reports [32–38], with only one experimental study in mice [30] and one cross-sectional study in humans [31]. Across these studies, cerebellar atrophy was generally mild to moderate and typically persisted despite clinical improvement [31–38]. Overall, these findings suggest that cerebellar atrophy appears to be a relatively uncommon finding in SLE, with limited information regarding its true prevalence, clinical significance, and progression. A summary of these articles can be found in supplementary Table 1. In a previous study from our group using voxel-based morphometry (VBM), structural differences in the form of significantly lower grey matter probability values in the bilateral lobule VIIIA could be seen in patients with SLE compared to HI, which showed a weak positive correlation with psychomotor speed [39].

Structural cerebellar alterations have not yet been investigated in larger SLE populations using direct volumetric measures, nor have they been related to NP manifestations or clinical symptoms in a broader spectrum, such as pain, fatigue, or depression. Therefore, we aim to compare cerebellar volumes, both total and subregional, in patients with SLE and healthy individuals (HI), and to relate the volumes with measures of clinical symptoms, in particular cognitive dysfunction, depression, fatigue, and pain in patients with SLE.

## Methods

### Patients and healthy individuals

Patients with a clinical diagnosis of SLE and meeting the SLICC classification criteria for SLE [11] were asked to participate in this study during follow-up visits at the outpatient rheumatology clinic at Skåne University Hospital, Lund. Although our patient group was recruited according to the SLICC criteria, we did not apply a categorical NPSLE classification in the present study, as there is growing evidence that neuronal damage in SLE may be present in all patients even in the absence of neuropsychiatric manifestations [40].

All patients included in this study were right-handed females with SLE, aged 18–55 years. Participants provided written informed consent, and those unable to provide consent or unable to perform cognitive testing or answer the clinical questionnaires were not included. The patient recruitment process and rheumatologic evaluation is previously described in detail by Zervides et al. [41]. HI participated as controls and were frequency-matched to the SLE patient group at a group level for age, sex, and right-handedness. Current, or past autoimmune disease, previously diagnosed neurological condition, or severe psychiatric condition were additional criteria for exclusion from the HI population.

Notably, several of the subjects included in this study were also part of a previously published study from our research group [39], which investigated VBM changes in cerebellar grey matter and their association with the psychomotor speed domain of Central Nervous System-Vital Signs (CNS-VS) in SLE patients. The current study revisits the cerebellum using direct volumetric measurement of the cerebellar grey and white matter and the individual cerebellar lobules. This study also includes analyses of the associations and correlations between volumes of different lobules and various clinical variables. This in contrast to previous VBM study that estimated grey matter atrophy without any correlation to different clinical variables other than cognitive parameters [39].

### Clinical data collection

Disease activity was assessed according to the SLE Disease Activity Index 2000 (SLEDAI-2 K) [42] and organ damage according to the SLICC Damage Index (SDI) [43]. Medication and medical history were recorded, and patients completed questionnaires regarding their education level; notably corresponding data were not available for HI. Given the prevalence of chronic pain in SLE patients [44], particularly fibromyalgia [45], pain was assessed using a self-reported Visual Analogue Scale 100 mm (VAS) for pain in the SLE group. Fatigue was assessed using both VAS for fatigue in the SLE group and Fatigue Severity Scale (FSS) in all participants, with significant fatigue defined as an FSS score equal to or above 36 [46]. Depressive symptoms were evaluated in all participants using a self-reported depression scale, the Montgomery–Åsberg Depression Rating Scale (MADRS-S) [47]. Depression severity was assessed using the MADRS-S scale, and the scores were binarized into normal MADRS-S scores (score 0–6) and MADRS-S scores reflecting depressive symptoms (score > 6) (for more details see Table 1).

### Cognitive testing

All participants were tested by a clinical psychologist using CNS-VS [48] to assess cognitive function. The CNS-VS test battery consists of seven parts that cover seven different single test cognitive domains and four multiple test cognitive domains which are combinations of the different basic domains [48]. In the present study, we chose five cognitive functions from the CNS-VS test output as they reflect the global neurocognitive scoring and represent the comprehensive view of cognitive function in SLE [48]: composite memory (combination of verbal memory and visual memory), psychomotor speed (combination of motor speed and processing speed), reaction time, complex attention (combination of reaction time, executive function, and simple attention), and cognitive flexibility (combination of reaction time and executive function).

Standardized cognitive domain scores produced by the CNS-VS software are normalized in relation to age-matched healthy controls to the expected mean of 100 and standard deviation of 15 [48]. A higher score is desirable, while a score of 79 and below (corresponding with a standard deviation of − 1.4) is equivalent of a moderate deficit in cognition where impairment is possible [48]. According to the validity indicator algorithm of CNS-VS [48], individual test results deemed as invalid were excluded, resulting in two excluded patients in the analyses related to cognitive function.

### Magnetic resonance imaging (MRI) acquisition and image processing

A 3 tesla (3T) MRI scanner (Siemens MAGNETOM Skyra, Erlangen, Germany) at Lund University Hospital was used to obtain T1-weighted Magnetization-Prepared RApid Gradient-Echo (MPRAGE) scans [1 mm isotropic voxel, echo time 2.54 ms/ repetition time 1900 ms/ inversion time 900 ms]. The scans were processed by CEREbellum Segmentation (CERES) volBrain version 1.0 [49] to obtain automatic total intracranial and cerebellar volumes, global and gray matter lobule volumes, and lobule segmentations. In this study, the term global volume refers to the combined volume of both grey matter and white matter. The term bilateral lobule refers to the combined volume of the left and right hemispheres of the same lobule.

CERES volBrain generated total intracranial volume (TIV) (in cm^3^), total cerebellar volume (TCV) (in cm3 and as percentage of TIV), and was also used for the segmentation and volume extraction of individual cerebellar lobules (in cm3 and as percentage of TIV) [49, 50]. Global and grey matter total cerebellar volumes, and global and grey matter lobule volumes (both in cm^3^ and as percentage of TIV) were provided by CERES volBrain. White matter volume of the cerebellum and of each lobule was extracted by subtracting the grey matter volume from the global volume for the total cerebellum and for each individual lobule.

In this study we chose to analyze both the bilateral volumes of the cerebellum and cerebellar lobules, to account for regional changes based on anatomy, as well as the left and right hemispheric volumes of the cerebellum and cerebellar lobules separately, due to the lateralization in the function of the cerebellum. All volumes (in cm^3^) in this study were normalized as percentages of the TCV, both to correct for the volume of the cerebellum and examine regional cerebellar proportions specifically.

**Fig. 1:**
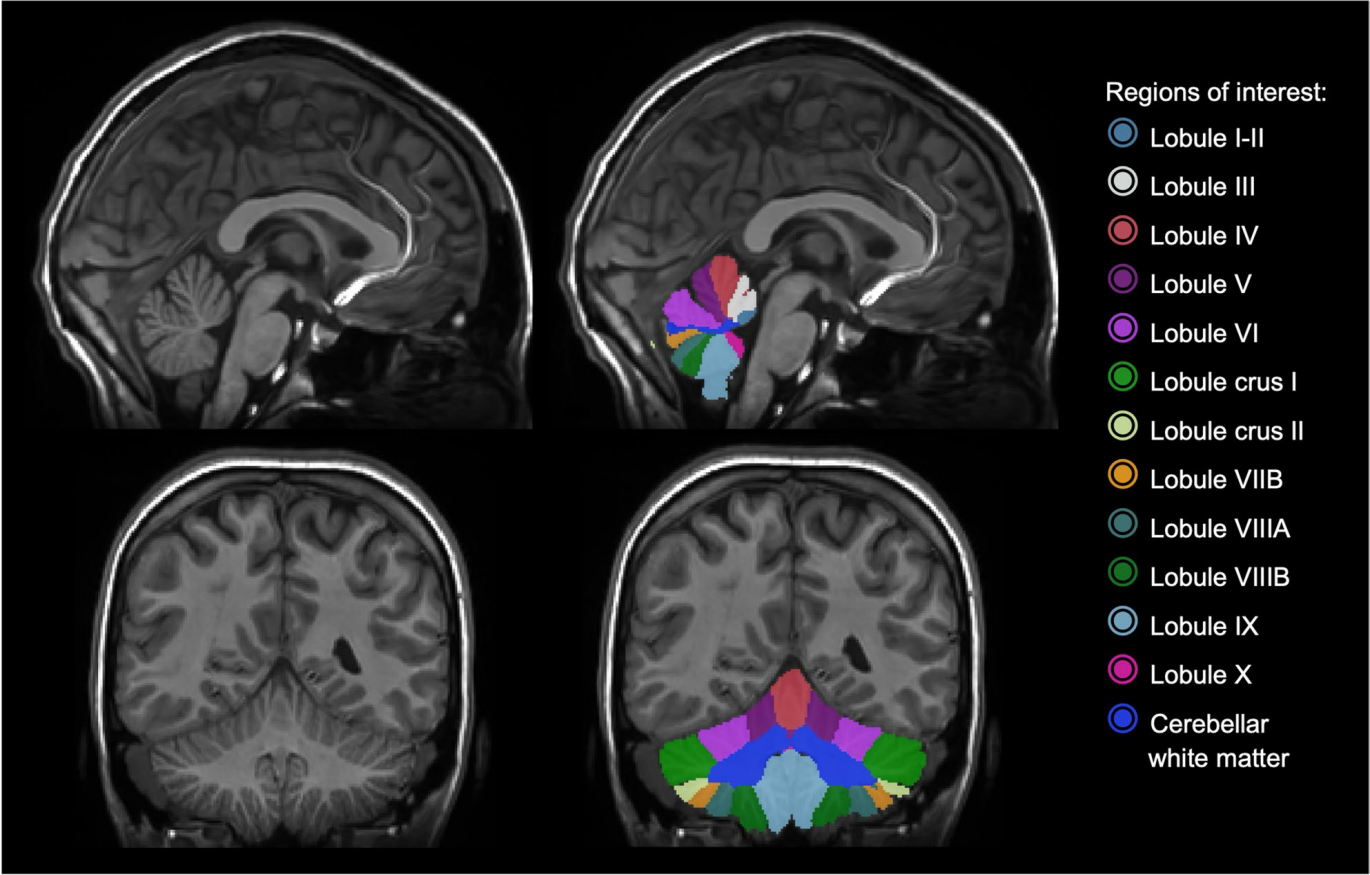
Example of CERES volBrain cerebellar segmentation in a randomly selected SLE patient

The segmentation of the 12 lobules by the CERES volBrain algorithm [49, 50] is based on the Schmahmann definition [51]: lobules I–II, lobule III, lobule IV, lobule V, lobule VI, crus I, crus II, lobule VIIB, lobule VIIIA, lobule VIIIB, lobule IX, and lobule X (Fig. 1). To ensure the validity of the extracted volumes, all segmentations were visually checked by a junior doctor (ZMN) and a senior neuroradiologist (PCS) with FreeView version 3.0, part of the FreeSurfer image analysis software version 7.1.1 (https://surfer.nmr.mgh.harvard.edu). All MR scans were inspected in the native space first in the sagittal plane, then in the axial plane followed by the coronal plane. Lobular segmentations were deemed accurate with only negligible segmentation errors compared to the total volume of each lobule; thus, no segmentation editing was required. This quality check was blinded to the disease status of the subjects.

### Statistical analyses

Descriptive analyses were performed to look at demographic differences between our SLE and HI cohorts. After checking for assumptions of normality, type III one-way Analyses of Covariance (ANCOVA) were performed to look at general differences in cerebral and cerebellar volumes between SLE patients and HI, adjusting for age. More detailed exploratory analyses to compare cerebellar lobular volumes between SLE patients and HI using type III one-way ANCOVA were performed, focusing on the volumes normalized as percentage of TCV and adjusting for age. We analyzed global, grey matter, and white matter volumes of each lobule in the right and left hemispheres separately, as well as in the bilateral lobules.

Focusing on the SLE group, we looked further into the relationship between volume and various clinical variables. Pearson’s partial correlation analyses were used to examine the relationship between cerebellar volumes normalized as percentage of TCV and continuous clinical data scores in the SLE patient group, adjusting for age. Clinical data scores consisted of continuous cognitive function scores measured using CNS-VS (additionally adjusting for education level), pain scores measured using VAS pain, fatigue scores measured using VAS fatigue and FSS. These analyses were performed in the cerebellar lobules that were significantly different in SLE patients compared to HI, aiming for detection of disease-specific correlations. We chose to focus on global volumes in our correlation analyses to minimize any possible influence of segmentation inaccuracies due to the structure of the cerebellum. Other potential confounding and disease-related variables, including disease duration, corticosteroid exposure, immunosuppressive treatment, and comorbidities, were considered. However, due to limitations in data availability, sample size, and risk of multicollinearity, these variables were not included as covariates in the main analyses. Additional exploratory analyses were performed within the SLE group to examine whether the cerebellar regions identified in the primary analyses were associated with potential confounding factors (education level, smoking status, hypertension, and antihypertensive treatment) or disease-related variables (disease duration, SLEDAI-2 K, SDI, corticosteroid treatment, and DMARDs) while correcting for age. These analyses were intended to provide additional context for interpretation of the primary findings rather than formal adjustment of the main analyses.

Additionally, comparisons of cerebellar lobular volumes normalized as percentage of TCV were performed between SLE patients with and without cognitive impairment or depressive symptoms using type III one-way ANCOVA, adjusting for age (and level of education for cognitive analyses). The clinical data subgroups were defined based on presence of cognitive impairment according to the CNS-VS definition of moderate deficit in cognition [48], and severity of depressive symptoms according to MADRS-S [47]. These analyses were also performed in the cerebellar lobules that were significantly different in SLE patients compared to HI.

Given the exploratory design of this study, no formal correction for multiple comparisons was applied. In all analyses, a p-value of < 0.05 was considered statistically significant. All analyses were performed using R Statistical Software version 4.4.0 and RStudio version 2024.04.2 + 764.

## Results

### Study population

A total of 97 participants met the inclusion criteria and were included in this study, 72 female SLE patients and 25 age- and sex-matched HI. On a group level, SLE disease activity was relatively low, with most patients displaying low SLEDAI-2 K scores (median score 2, range 0–18). However, formal remission criteria such as Lupus Low Disease Activity State (LLDAS) and Definition of Remission in SLE (DORIS) were not used [52, 53]. Consequently, our findings primarily reflect cerebellar alterations in a relatively well-treated and clinically stable SLE population and may not be generalizable to patients with higher disease activity. As expected, SLE patients displayed significantly increased levels of fatigue and depressive scores compared to HI. At the time of the study, 59% of the patients were on non-antimalarial disease-modifying anti-rheumatic drugs (DMARDs), 79% on antimalarial DMARDs (hydroxychloroquine), 30.6% were on anti-hypertensive medication, and 79% were on corticosteroid medication, with a median daily dose of 5 mg prednisolone (range 0–25 mg). Clinical characteristics of the study population are presented in Table 1.

Exploratory analyses were performed to examine whether the cerebellar regions identified in the primary analyses (bilateral lobules IV and VIIB) were associated with potential confounding factors, including education level, smoking status, hypertension, and antihypertensive treatment, as well as disease-related variables, including disease duration, SLEDAI-2 K, SDI, corticosteroid treatment, and antimalarial or non-antimalarial DMARD treatment (see supplementary Table 2). No significant associations between cerebellar volumes and potential confounders were observed.

**Table 1:** Demographics and clinical characteristics of the study cohort

		SLE patients	HI
**Number**		72	25
**Clinical data**			
**Age [years]**,** median (range)**		37.5 (18–51)	40 (23–52)
**Smoking (ever)**,** n (%)**		45 (62.5)	–
**Education level**,** n (%)**	**0**, comprehensive school (year 1–9) or lower	1 (1.4)	–
	**1**, completed upper secondary school (year 10–12)	34 (47.9)	–
	**2**, completed higher educational levels	36 (50.7)	–
**MADRS-S score**,** mean ± SD**		12.71 ± 9.41	2.48 ± 2.93
**MADRS-S groups**,** n (%)**	**1**, normal, score of 0–6	22 (30.6)	22 (88.0)
	**2**, mild, score of 7–19	35 (48.6)	3 (12.0)
	**3**, moderate, score of 20–34	13 (18.1)	0 (0.0)
	**4**, severe, score of more than 34	2 (2.8)	0 (0.0)
**FSS score**,** mean ± SD**		41.45 ± 15.04	21.20 ± 8.56
**FSS clinically significant (value over 36)**,** n (%)**		49 (69.0)	1 (4.0)
**VAS fatigue**,** mean ± SD**		55.82 ± 31.09	–
**VAS pain**,** mean ± SD**		32.24 ± 28.97	–
**Cognitive dysfunction**,** n (%)**	± 1.4 SD, in one domain	36 (52.2)	10 (40.0)
	± 1.4 SD, in two or more domains	20 (29.0)	3 (12.0)
**Disease duration [years]**,** median (range)**		10 (0–32)	–
**SLEDAI-2 K**,** median (range)**		2 (0–18)	–
**SDI**,** median (range)**		0 (0–5)	–
**Hypertension**,** n (%)**		15 (24.2)	–
**Diabetes**,** n (%)**		0 (0)	–
***Medication***			
**DMARDs**,** n (%)**	**Ongoing non-antimalarial**	42 (59.2)	–
	**Ongoing antimalarial (hydroxychloroquine)**	57 (79.2)	–
**Ongoing antihypertensive treatment**,** n (%)**		22 (30.6)	–
**Ongoing corticosteroid treatment**,** n (%)**		57 (79.2)	–
**Corticosteroid daily dose (mg/day)**,** median (range)**		5 (0–25)	–

Abbreviations: SLE: Systemic lupus erythematosus; HI: healthy individuals; n: number; MADRS-S: Montgomery–Åsberg depression rating scale self-reported; SD: standard deviation; FSS: fatigue severity scale; VAS: visual analogue scale; SLEDAI-2 K: SLE disease activity index 2000; SDI: SLICC damage index; DMARDs: disease-modifying antirheumatic drugs

### Global brain volumes in SLE compared to HI

No significant group differences were found in total intracranial volume, total global cerebral and cerebellar volume, or the cerebellum-cerebrum ratio between SLE patients and HI (see supplementary Table 3).

### Cerebellar lobular volumes in SLE patients compared to HI

**Fig. 2:**
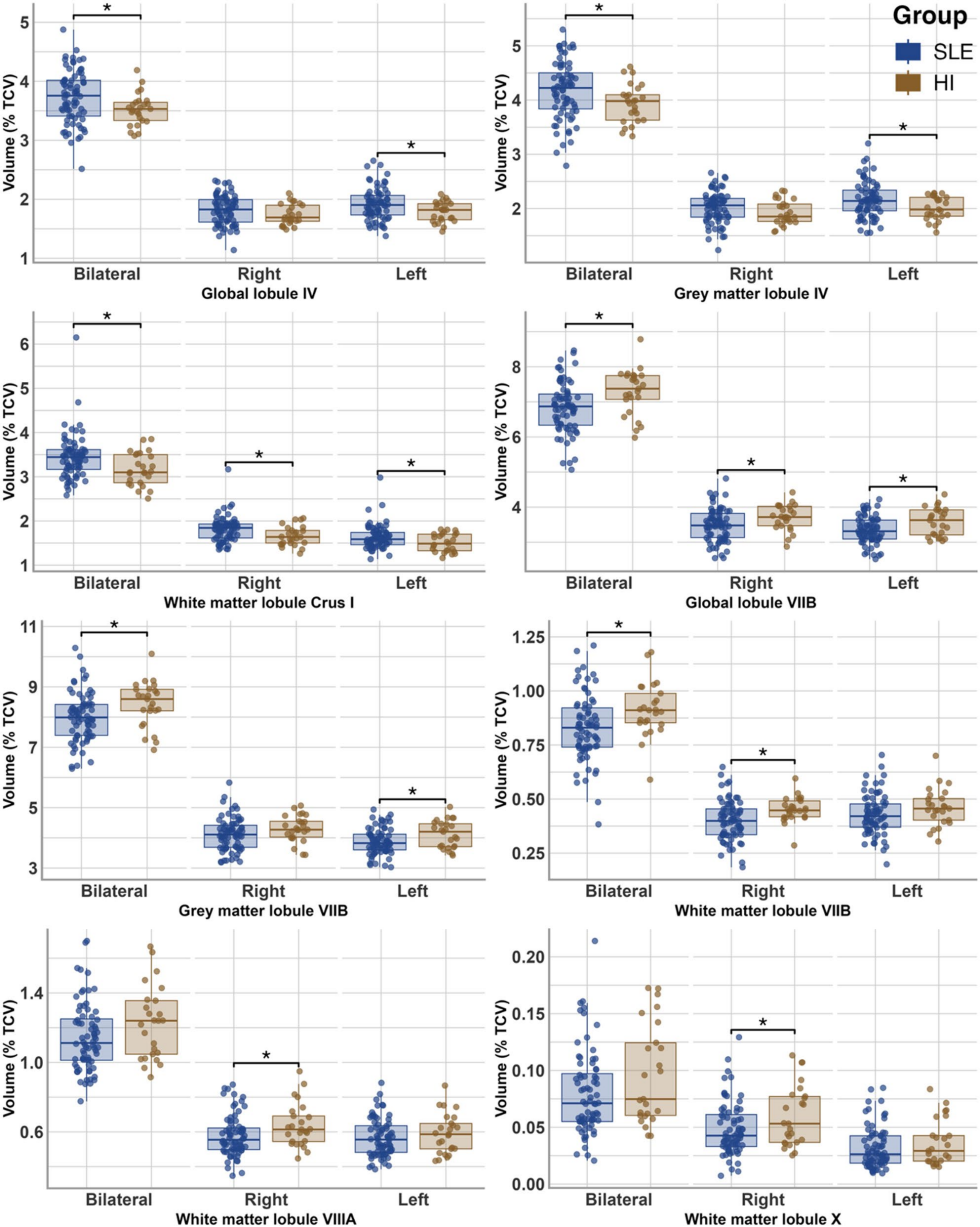
Cerebellar volume differences between SLE patients and HI. Asterisk represents *p* < 0.05

Next, we analyzed global, grey matter, and white matter volumes of individual cerebellar lobules, normalized as percentages of TCV, comparing SLE patients and HI. Significantly larger volumes were observed in bilateral lobule IV (global volume: *p* = 0.02; grey matter volume: *p* = 0.03) and in left lobule IV (global volume: *p* = 0.02; grey matter volume: *p* = 0.03) of the cerebellum in SLE patients compared to HI. Significantly smaller volumes were observed in bilateral lobule VIIB (global volume: *p* = 0.008; grey matter volume: *p* = 0.02; white matter volume: *p* = 0.01), right lobule VIIB (global volume: *p* = 0.04; white matter volume: *p* = 0.009), and left lobule VIIB (global volume: *p* = 0.01; grey matter volume: *p* = 0.01) of the cerebellum in SLE patients compared to HI. In addition, significantly smaller white matter volumes were observed in right lobule VIIIA (*p* = 0.04) and right lobule X (*p* = 0.04) while significantly larger white matter volumes were observed in lobule crus I (bilateral: *p* = 0.01; right: *p* = 0.01; left: *p* = 0.01) in SLE patients compared to HI. No significant differences were found in the remaining cerebellar lobules. Significant results are shown in Table 2; Fig. 2, and the full set of comparisons are displayed in supplementary Table 4.

**Table 2:** Significant differences in cerebellar volumes between SLE patients and HI

Region-of-interest	Cerebellar global* volume as a percentage of total cerebellar volume			Cerebellar grey matter volume as a percentage of total cerebellar volume			Cerebellar white matter volume as a percentage of total cerebellar volume		
	SLE patients (%)	Healthy individuals (%)	*p*–value	SLE patients (%)	Healthy individuals (%)	*p*–value	SLE patients (%)	Healthy individuals (%)	*p*–value
	Estimated means ± SE	Estimated means ± SE		Estimated means ± SE	Estimated means ± SE		Estimated means ± SE	Estimated means ± SE	
Number	72	25		72	25		72	25	
**Lobule IV**									
Bilateral lobule IV	3.73 ± 0.45	3.52 ± 0.26	**0.02**	4.17 ± 0.53	3.93 ± 0.35	**0.03**	0.59 ± 0.13	0.54 ± 0.09	0.12
Left lobule IV	1.91 ± 0.26	1.78 ± 0.17	**0.02**	2.16 ± 0.33	2.00 ± 0.21	**0.03**	0.29 ± 0.06	0.26 ± 0.05	0.10
**Lobule crus I**									
Bilateral lobule crus I	21.03 ± 2.18	20.14 ± 1.64	0.06	23.35 ± 2.37	22.43 ± 1.73	0.07	3.44 ± 0.50	3.16 ± 0.37	**0.01**
Right lobule crus I	10.61 ± 1.16	10.15 ± 0.82	0.07	11.68 ± 1.27	11.22 ± 0.84	0.09	1.81 ± 0.28	1.66 ± 0.22	**0.01**
Left lobule crus I	10.42 ± 1.10	9.99 ± 0.90	0.08	11.67 ± 1.19	11.21 ± 0.97	0.08	1.63 ± 0.26	1.50 ± 0.20	**0.03**
**Lobule VIIB**									
Bilateral lobule VIIB	6.84 ± 0.72	7.28 ± 0.65	**0.008**	7.99 ± 0.81	8.42 ± 0.75	**0.02**	0.82 ± 0.15	0.91 ± 0.12	**0.01**
Right lobule VIIB	3.49 ± 0.46	3.70 ± 0.36	**0.04**	4.11 ± 0.53	4.30 ± 0.43	0.11	0.40 ± 0.09	0.45 ± 0.05	**0.009**
Left lobule VIIB	3.34 ± 0.38	3.57 ± 0.39	**0.01**	3.87 ± 0.42	4.12 ± 0.45	**0.01**	0.42 ± 0.09	0.46 ± 0.08	0.12
**Lobule VIIIA**									
Right lobule VIIIA	4.32 ± 0.49	4.52 ± 0.50	0.08	4.97 ± 0.57	5.14 ± 0.58	0.22	0.58 ± 0.11	0.63 ± 0.12	**0.04**
**Lobule X**									
Right lobule X	0.49 ± 0.05	0.49 ± 0.06	0.94	0.59 ± 0.07	0.58 ± 0.09	0.27	0.04 ± 0.02	0.06 ± 0.02	**0.04**

* Global = both grey and white matter

### Cerebellar lobular volumes and measures of cognition, depression, pain and fatigue

**Fig. 3:**
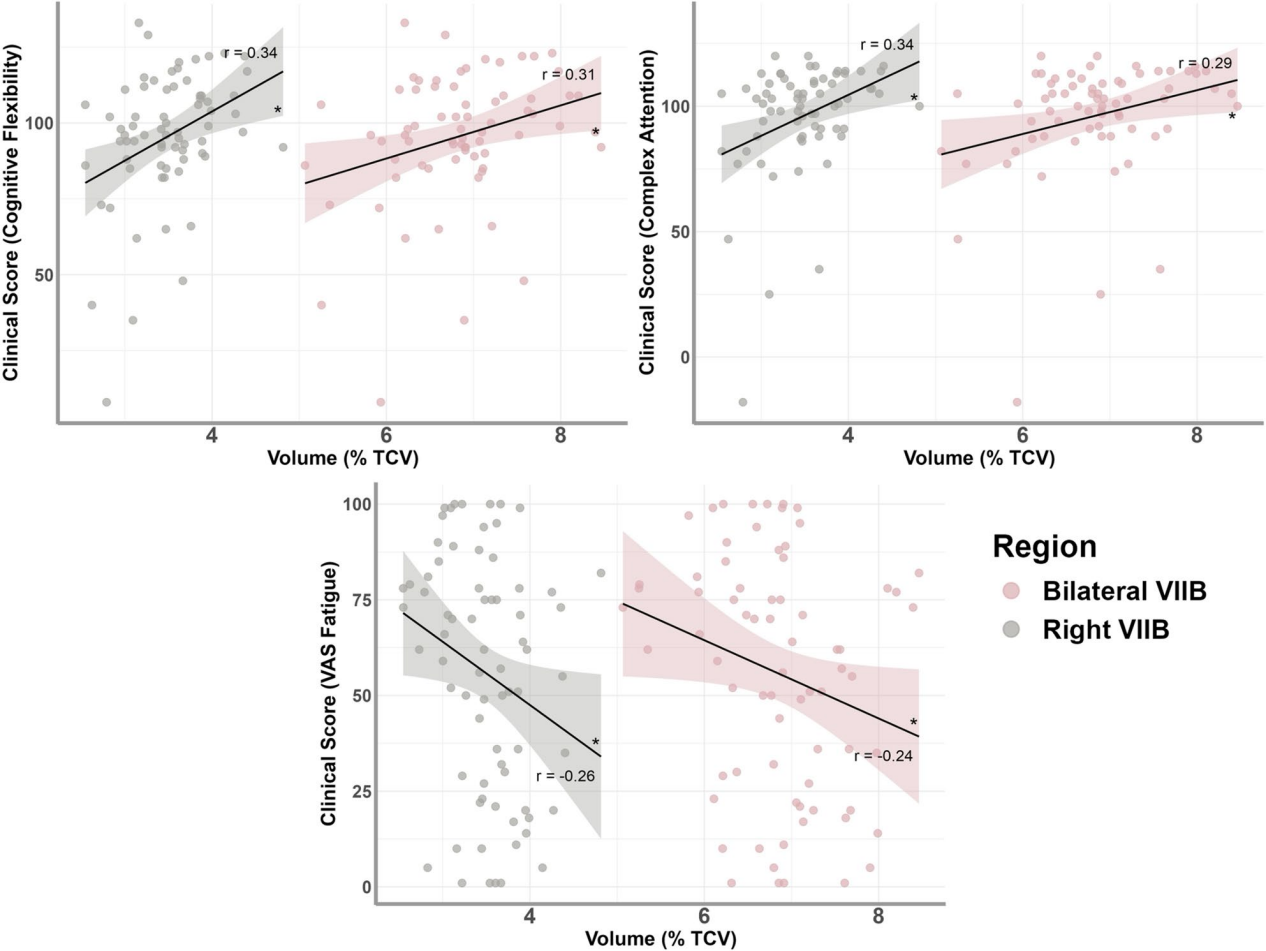
Partial correlations between cerebellar lobular volumes and clinical scores. Asterisk represents *p* < 0.05

Smaller global volumes in bilateral lobule VIIB (combined left and right hemisphere) and specifically in right lobule VIIB were associated with worse complex attention (*r* = 0.26, *p* = 0.03; *r* = 0.31, *p* = 0.01, respectively) and cognitive flexibility (*r* = 0.28, *p* = 0.02; *r* = 0.31, *p* = 0.008, respectively). Smaller global volumes in bilateral and right lobule VIIB, normalized as percentages of TCV, were also associated with higher VAS fatigue scores (*r* = -0.23, *p* = 0.04; *r* = -0.26, *p* = 0.02, respectively). No significant associations were found in the remaining cerebellar global lobular volumes. Significant results are shown in Table 3; Fig. 3, and the full set of comparisons are displayed in supplementary Table 5.

Cerebellar global lobular volumes did not differ between groups when cognitive function and depressive scores were analyzed as categorical variables. The full set of comparisons is presented in supplementary Table 6.

**Table 3:** Significant correlations between cerebellar volumes as a percentage of TCV and continuous scores of cognitive function and fatigue

Region-of-interest	Cerebellar global* volume as a percentage of total cerebellar volume	
	Partial correlation coefficient (*r*)	*p*-value
**Complex attention**		
Number	67	
Bilateral lobule VIIB	0.26	**0.03**
Right lobule VIIB	0.31	**0.01**
**Cognitive flexibility**		
Number	68	
Bilateral lobule VIIB	0.28	**0.02**
Right lobule VIIB	0.31	**0.008**
**Fatigue VAS score**		
Number	72	
Bilateral lobule VIIB	-0.23	**0.04**
Right lobule VIIB	-0.26	**0.02**

* Global = both grey and white matter

## Discussion

To our knowledge, this is one of the first studies to systematically investigate cerebellar subregional volumes in a relatively large group of patients with SLE using a lobular volumetric approach. Previous neuroimaging studies in SLE have primarily focused on supratentorial brain regions, while cerebellar involvement has comparatively received little attention despite the growing recognition of the cerebellum’s role in cognition, affective regulation, and fatigue. In this study, we found selective differences in cerebellar lobular volumes between patients with SLE and HI when normalized to total cerebellar volume. Global lobule VIIB of the cerebellum was smaller in SLE patients compared to HI and global lobule IV of the cerebellum was larger in SLE patients compared to HI. The restriction of findings to specific regions suggests that SLE affects the cerebellum in a regional selective manner, rather than causing generalized cerebellar atrophy. Within the SLE group, smaller global lobule VIIB volumes correlated with worse cognitive function in the complex attention and cognitive flexibility domains and with worse fatigue scores.

Lobule VIIB belongs to the so-called “cognitive cerebellum”, which is connected to the limbic system and associative cortex [23]. This lobule has been shown to be involved in language processing, executive functions (such as working memory, planning, organizing, and strategy formation), and the processing of negative input [24, 54, 55]. These functions align with our findings showing significant correlations between smaller global lobule VIIB volumes and poorer cognitive performance in complex attention and cognitive flexibility, with executive function contributing to both domains. Our findings are consistent with those seen in patients with cerebellar cognitive affective syndrome, which is associated with damage to lobules VIIB and IX, and shows deficits in executive function amongst other cognitive domains [24]. Lobule VIIB involvement has also been reported in multiple sclerosis, where atrophy of posterior cerebellum lobules, including lobule VIIB, have been described [56]. In addition, larger volumes of lobule VII have been associated with better motor and cognitive performance in multiple sclerosis [57]. More specifically, grey matter volume loss in lobule VIIB, particularly in the right hemisphere, has been reported in multiple sclerosis [58], whereas in our present study we observed significantly smaller left lobule VIIB grey matter volumes in patients with SLE.

Smaller bilateral lobule VIIB volume within the SLE group was also associated with greater fatigue severity in patients with SLE. This finding is in line with emerging evidence suggesting that the cerebellum plays a role in fatigue perception and fatiguability [27]. To our knowledge, similar lobule-specific correlations with fatigue have not previously been reported in SLE or in other conditions such as multiple sclerosis or other neuroinflammatory or systemic diseases. While several cerebellar and vermal regions have been linked to pain perception and processing, including lobules IV, V, VI, VIIA (crus I and crus II), and VIIB [24, 54, 59–62], we found no significant correlations between cerebellar lobular volumes/lobule VIIB volume and VAS pain scores in our study.

The findings of larger global and grey matter volume in lobule IV and larger white matter volume in crus I in patients with SLE were unexpected. Lobule IV belongs to the so-called “primary sensorimotor cerebellum” in the anterior cerebellum, whereas crus I belongs to lobule VIIA and is part of the cognitive cerebellum [23]. Several possible explanations may underlie these findings of increased volume. Speculating, a possible explanation is structural remodeling, where compensatory regional volume increase can occur in response to regional volume loss elsewhere or in response to affected functions in said region [63, 64]. The theory of cerebellar remodeling is based on structural neuroplasticity and has been reported in conditions other than neuroinflammation, including stroke, traumatic brain injury, or other pathology to the cerebellum [65–67]. However, these interpretations remain speculative in the context of the present cross-sectional study. Alternative explanations such as low-grade inflammatory processes, vascular-related changes, or transient tissue alterations not detectable in conventional MRI should also be considered. It is also possible that these findings are spurious or unspecific to SLE, as involvement of cerebellar lobule IV or crus I has not previously been reported in SLE, and the significance of these findings therefore remains unclear.

The predominance of findings in the right cerebellar hemisphere may relate to the known structural and functional asymmetry between the left and right cerebellar hemispheres [24, 54, 55]. Although the left cerebellar hemisphere tends to be slightly larger than the right [23], such baseline asymmetry is unlikely to account for the observed volume differences between patients with SLE and HI. The lateralization observed in the correlation analyses is more plausibly explained by the functional lateralization in the cerebellum, as language tasks are typically right-lateralized and spatial tasks are left-lateralized in the cerebellum, reflecting the contralateral cortico-cerebellar connections [24, 54, 55].

There is an overlap between the functions of the different lobules. Recent studies on the functional organization of the cerebellum showed that individual lobules are involved in multiple functions, with different activation patterns depending on the task [68, 69]. We found lobular volume differences associated with clinical symptoms in patients with SLE. Although the mechanisms behind cerebellar involvement in SLE remain unclear and were not directly investigated in the present study, many theories have been suggested. These mechanisms possibly mirror cerebral involvement, where ischemic infarctions, vasogenic edema, small vessel vasculopathies, or immunoglobulin-, cytokine-, and antibody-mediated cerebral dysfunction are the usual culprits [30, 32, 70, 71]. However, it is unclear if these pathologies are directly caused by damage to the cerebellum itself or due to disruption in the pathways connecting the cerebellum to the cerebral regions responsible for these functions [23].

Structural cerebellar changes in the form of atrophy appear to be unrelated to both clinical outcomes and treatment response in SLE. In a few case reports of SLE with cerebellar involvement, some patients with SLE showed no symptom reversal after treatment [32, 36, 37], while others showed clinical improvement despite persistent atrophy on MRI [33–35, 38]. However, the patients described in those studies are very different from our study group of patients with SLE with low disease activity according to SLEDAI-2 K.

Our findings are partly consistent with previous neuroimaging studies reporting cerebellar involvement in SLE, such as the previous VBM study from our research group [39] and a more recent VBM study in patients with childhood-onset SLE [72], supporting the growing recognition of cerebellar involvement in SLE. These findings may also have implications for future neuroimaging studies in SLE, motivating the inclusion of dedicated cerebellar acquisition and analysis strategies, including connectivity analyses and cerebellum-focused functional MRI. The current findings support continued investigation of cerebellar involvement in SLE and may help guide future studies aimed at identifying imaging biomarkers associated with cognitive dysfunction and fatigue.

Our study has some limitations. As an exploratory analysis aiming to identify potential patterns of cerebellar involvement in SLE, no formal correction for multiple comparisons was applied. While this approach may reduce the risk of type II error and allow detection of potential regional effects for future investigation, it also increases the risk of type I error. Therefore, the reported regional differences should be interpreted as exploratory and in need of confirmation in future studies. While we included a relatively large number of participants, the power of the study may still be limited. Our results are not generalizable to male patients with SLE and patients outside the age range of 18–55 years. Furthermore, the study group consists mainly of patients with Caucasian descent. Due to methodological limitations, adjustment for potential confounders was limited. Information on cumulative corticosteroid exposure was not available, and only treatment status and current daily dose were recorded. Because cumulative exposure, rather than current daily dose, has been linked to focal brain atrophy in SLE [73–75], we could not adequately adjust for potential long-term effects of corticosteroid treatment. Additional exploratory sensitivity analyses using the available corticosteroid variables (current treatment status and daily dose) were performed and are presented in the supplementary material, however these measures cannot be considered adequate proxies for cumulative corticosteroid exposure. Other potential confounders and disease-related variables, including such as hypertension, antihypertensive treatment, DMARDs, smoking, and education level were included in the additional sensitivity analyses and showed no significant associations with the cerebellar regions that differed between SLE patients and healthy individuals. These findings suggest that the observed cerebellar volume differences are not explained by the available disease-related variables or measured potential confounders, although the analyses should be interpreted cautiously given the exploratory nature of the study and the limited sample size. Other cardiovascular risk factors such as dyslipidemia, were not available and could therefore not be evaluated. Another possible confounder is education level, which was not available for the healthy individual group, and it is therefore possible that a potential group difference in educational level may have confounded our results. Additionally, the cross-sectional approach of this study prohibits conclusions on how changes occur over time, as well as cause and effect relationships. Cerebellar regions such as the vermis, paravermal areas, and the deep cerebellar nuclei are not defined separately by the CERES volBrain algorithm but are rather included within other cerebellar lobules [66], therefore prohibiting specific analyses focused on these subregions.

## Conclusion

The findings suggest that the cerebellum, and particularly cerebellar lobule VIIB, may contribute to both cognitive dysfunction and fatigue in patients with systemic lupus erythematosus. Further research is warranted to better understand cerebellar involvement in SLE, including functional and longitudinal studies.

## Supplementary Information

Below is the link to the electronic supplementary material.

Supplementary Material 1

Supplementary Material 2

Supplementary Material 3

Supplementary Material 4

Supplementary Material 5

Supplementary Material 6

## Data Availability

The datasets used and/or analyzed during the current study are available from the corresponding author on reasonable request.

## Abbreviations

SLE: Systemic Lupus Erythematosus
NP: Neuropsychiatric
NPSLE: Neuropsychiatric Systemic Lupus Erythematosus
ACR: American College of Rheumatology
SLICC: Systemic Lupus International Collaborating Centers
VBM: Voxel-Based Morphometry
HI: Healthy Individuals
CNS-VS: Central Nervous System-Vital Signs
SLEDAI-2K: SLE Disease Activity Index 2000
SDI: SLICC Damage Index
VAS: Visual Analogue Scale
FSS: Fatigue Severity Scale
MADRS-S: Montgomery–Åsberg Depression Rating Scale self-reported
3T: 3 Tesla
MPRAGE: Magnetization-Prepared RApid Gradient-Echo
CERES: CEREbellum Segmentation
TIV: Total Intracranial Volume
TCV: Total Cerebellar Volume
ANCOVA: ANalyses of COVAriance
DMARD: Disease-Modifying Anti-Rheumatic Drug
Global volume: volumes of both cerebellar white and gray matter
