# Supplementary material for: Cerebellar volume alterations are associated with cognitive dysfunction and fatigue in patients with systemic lupus erythematosus
Source: BMC Rheumatol. 2026 Jul 2;10:56. doi: 10.1186/s41927-026-00671-7 (PMC13335149; doi:10.1186/s41927-026-00671-7)
Supplement: Supplementary file 1 — Supplementary Material 1 [file 41927_2026_671_MOESM1_ESM.docx]

**Supplementary table 1**: Summary of articles discussing cerebellar volumes in SLE patients.

| **Article (year)** | **Study type** | **Participants** | **Imaging findings** | **Reversal of symptoms and/or imaging findings** | **Additional information** |
| --- | --- | --- | --- | --- | --- |
| Sled et al (2009) (30) | Experimental | 81 MRL/lpr mice, 47 MRL+/+ mice controls | MRI: widespread brain volume reduction in MRL/lpr mice compared to controls. Cerebellar peduncles & arbor vitae significantly smaller, cerebellar cortex volume reduction not significant. |  | Volume reduction found in areas bordering the ventricular system. |
| Sibbit et al (1989) (31) | Cross-sectional | 21 SLE (10-62 years), sex not specified | CT: atrophy in 2/21 cases.  MRI: atrophy in 7/21 cases. |  | Atrophy in CT was associated with symptoms of headache and organic brain syndrome. Atrophy on MRI was associated with longstanding SLE, age, recurrent infarction. Generalized atrophy more common in disease ≥ 5 years (6/8 cases). |
| Shimomura et al (1993) (32) | Letter to editor | 1 case (47 years, female, prior SLE) | CT: no abnormality  MRI: initial showed no abnormality, second showed cerebellar atrophy. | No symptom improvement. | Autoantibodies against Purkinje-cell and 75 kiloDalton protein in the cerebellar cortex were found. |
| Al-Arfaj et al (1995) (33) | Case report | 1 case (40 years, female, prior SLE) | CT: mild bilateral cerebellar atrophy with moderately severe vermal atrophy.  MRI at later timepoint: marked cerebellar atrophy. | Partial symptom improvement after 8 years, but MRI atrophy persisted. | Cerebellar atrophy on MRI showed no evidence of demyelination. |
| Manto et al (1996) (34) | Case report | 1 case (27 years, female, no prior SLE) | MRI: pancerebellar atrophy without signal intensity changes within the cerebellum. | Partial symptom improvement over 10 days; mild tremor persisted at 8 months. MRI atrophy was unchanged. |  |
| Chattopadhyay et al (2011) (35) | Case report | 1 case (22 years, female, prior SLE) | MRI: mild cerebral and cerebellar atrophy, corpus callosal atrophy. | Full symptom resolution in 10 days. | Symptoms appeared 7 days after SLE diagnosis/ treatment. |
| Ghosh et al (2014) (36) | Case report | 1 case (22 years, female, no prior SLE) | MRI & CT: generalized cerebellar atrophy | No symptom improvement at 3 months. | Symptoms suggest a disorder affecting both cerebellar hemispheres and vermis. MRI findings referenced, but CT images were shown in the article. |
| Fujimori et al (2016) (37) | Case report | 1 case (35 years, female, prior SLE) | MRI: at admission and follow-ups showed no atrophy. At 17 month follow up revealed diminished lesion and left-dominant cerebellar hemisphere atrophy. | Symptoms fluctuated, worsening over time. | Progressive cerebellar atrophy despite treatment, suggesting ischemic rather than infectious or demyelinating pathology. |
| Casciato et al (2018) (38) | Case report | 1 case (42F, no prior SLE) | MRI: marked left cerebellar hemisphere atrophy involving the vermis. | Slight symptom improvement at 6 months. | Patient refused follow-up/treatment. |
| **Abbreviations**: MRL/lpr: Murphy Roths Large/lymphoproliferation; SLE: Systemic Lupus Erythematosus. | | | | | |
