# Supplementary material for: Cerebellar volume alterations are associated with cognitive dysfunction and fatigue in patients with systemic lupus erythematosus
Source: BMC Rheumatol. 2026 Jul 2;10:56. doi: 10.1186/s41927-026-00671-7 (PMC13335149; doi:10.1186/s41927-026-00671-7)
Supplement: Supplementary file 2 — Supplementary Material 2 [file 41927_2026_671_MOESM2_ESM.docx]

**Supplementary table 2**: Sensitivity analysis of possible confounders and disease-related variables related to volume.

| **Cerebellar global* volume as a percentage of total cerebellar volume** | **Continuous potential confounders** | **n** | **Partial correlation coefficient (ρ)** | **p-value** |
| --- | --- | --- | --- | --- |
| Bilateral lobule IV | Education level | 71 | 0.002 | 0.98 |
|  | Cigarette per day | 71 | 0.05 | 0.68 |
| Bilateral lobule VIIB | Education level | 71 | 0.02 | 0.82 |
|  | Cigarette per day | 71 | -0.09 | 0.41 |
| **Cerebellar global* volume as a percentage of total cerebellar volume** | **Binary potential confounders** | **n** | **Unstandardized Beta (β)** | **p-value** |
| Bilateral lobule IV | Hypertension | 62 | -0.002 | 0.98 |
|  | Ongoing antihypertensive treatment | 72 | -0.03 | 0.76 |
|  | Smoking (ever) | 70 | 0.11 | 0.31 |
| Bilateral lobule VIIB | Hypertension | 62 | -0.31 | 0.16 |
|  | Ongoing antihypertensive treatment | 72 | -0.36 | 0.05 |
|  | Smoking (ever) | 70 | -0.05 | 0.74 |
| **Cerebellar global* volume as a percentage of total cerebellar volume** | **Continuous disease-related variables** | **n** | **Partial correlation coefficient (ρ)** | **p-value** |
| Bilateral lobule IV | Disease duration | 72 | 0.10 | 0.38 |
|  | SLEDAI-2K | 72 | 0.07 | 0.52 |
|  | SDI | 72 | 0.01 | 0.87 |
|  | Corticosteroid daily dose (mg/day) | 72 | 0.004 | 0.97 |
| Bilateral lobule VIIB | Disease duration | 72 | 0.09 | 0.41 |
|  | SLEDAI-2K | 72 | 0.01 | 0.90 |
|  | SDI | 72 | -0.11 | 0.33 |
|  | Corticosteroid daily dose (mg/day) | 72 | -0.04 | 0.69 |
| **Cerebellar global* volume as a percentage of total cerebellar volume** | **Binary disease-related variables** | **n** | **Unstandardized Beta (β)** | **p-value** |
| Bilateral lobule IV | Ongoing corticosteroid treatment | 72 | 0.07 | 0.57 |
|  | Ongoing non-antimalarial | 71 | 0.07 | 0.46 |
|  | Ongoing antimalarial (hydroxychloroquine) | 72 | -0.09 | 0.46 |
| Bilateral lobule VIIB | Ongoing corticosteroid treatment | 72 | -0.10 | 0.63 |
|  | Ongoing non-antimalarial | 71 | -0.06 | 0.71 |
|  | Ongoing antimalarial (hydroxychloroquine) | 72 | 0.16 | 0.43 |
| * Global = both grey and white matter.  **Abbreviations**: SLEDAI-2K: SLE disease activity index 2000; SDI: SLICC damage index. | | | | |
