# Supplementary material for: Cerebellar volume alterations are associated with cognitive dysfunction and fatigue in patients with systemic lupus erythematosus
Source: BMC Rheumatol. 2026 Jul 2;10:56. doi: 10.1186/s41927-026-00671-7 (PMC13335149; doi:10.1186/s41927-026-00671-7)
Supplement: Supplementary file 3 — Supplementary Material 3 [file 41927_2026_671_MOESM3_ESM.docx]

**Supplementary table 3**: Differences in global brain volumes between SLE patients and HI.

| **Region-of-interest** | **SLE patients** | **Healthy individuals** | **p–value** |
| --- | --- | --- | --- |
|  | Estimated means ± SE | Estimated means ± SE |  |
| Number | 72 | 25 |  |
| Total intracranial volume (cm3) | 1363.61 ± 80.27 | 1359.15 ± 119.54 | 0.83 |
| Total global* cerebral volume (cm3) | 1236.00 ± 77.66 | 1228.31 ± 113.87 | 0.70 |
| Total global* cerebellar volume (cm3) | 127.61 ± 13.05 | 130.84 ± 10.74 | 0.27 |
| Total grey matter cerebellar volume (cm3) | 96.04 ± 10.28 | 98.99 ± 8.50 | 0.19 |
| Total white matter cerebellar volume (cm3) | 31.56 ± 3.42 | 31.84 ± 2.97 | 0.71 |
| Cerebellum-to-cerebrum volume ratio | 0.10 ± 0.01 | 0.10 ± 0.01 | 0.18 |
| * Global = both grey and white matter. | | | |
