# Supplementary material for: Cerebellar volume alterations are associated with cognitive dysfunction and fatigue in patients with systemic lupus erythematosus
Source: BMC Rheumatol. 2026 Jul 2;10:56. doi: 10.1186/s41927-026-00671-7 (PMC13335149; doi:10.1186/s41927-026-00671-7)
Supplement: Supplementary file 4 — Supplementary Material 4 [file 41927_2026_671_MOESM4_ESM.docx]

**Supplementary table 4**: Differences in cerebellar volumes between SLE patients and HI using ANCOVA.

| **Region-of-interest** | **Cerebellar global* volume as a percentage of total cerebellar volume** | | | **Cerebellar grey matter volume as a percentage of total cerebellar volume** | | | **Cerebellar white matter volume as a percentage of total cerebellar volume** | | |
| --- | --- | --- | --- | --- | --- | --- | --- | --- | --- |
|  | **SLE patients (%)** | **Healthy individuals (%)** | **p–value** | **SLE patients (%)** | **Healthy individuals (%)** | **p–value** | **SLE patients (%)** | **Healthy individuals (%)** | **p–value** |
|  | Estimated means ± SE | Estimated means ± SE |  | Estimated means ± SE | Estimated means ± SE |  | Estimated means ± SE | Estimated means ± SE |  |
| Number | 72 | 25 |  | 72 | 25 |  | 72 | 25 |  |
| **Lobule I-II** | | | | | | | | | |
| Bilateral lobule I-II | 0.09 ± 0.03 | 0.10 ± 0.03 | 0.70 | 0.06 ± 0.03 | 0.07 ± 0.02 | 0.89 | 0.04 ± 0.01 | 0.04 ± 0.02 | 0.59 |
| Right lobule I-II | 0.05 ± 0.01 | 0.05 ± 0.02 | 0.80 | 0.03 ± 0.01 | 0.03 ± 0.01 | 0.81 | 0.02 ± 0.01 | 0.02 ± 0.01 | 0.78 |
| Left lobule I-II | 0.04 ± 0.01 | 0.05 ± 0.02 | 0.35 | 0.03 ± 0.01 | 0.03 ± 0.01 | 0.67 | 0.02 ± 0.008 | 0.02 ± 0.01 | 0.17 |
| **Lobule III** | | | | | | | | | |
| Bilateral lobule III | 1.10 ± 0.20 | 1.09 ± 0.17 | 0.87 | 0.99 ± 0.22 | 0.96 ± 0.16 | 0.56 | 0.35 ± 0.08 | 0.36 ± 0.08 | 0.62 |
| Right lobule III | 0.55 ± 0.11 | 0.55 ± 0.10 | 0.80 | 0.50 ± 0.11 | 0.47 ± 0.10 | 0.38 | 0.17 ± 0.04 | 0.18 ± 0.04 | 0.37 |
| Left lobule III | 0.54 ± 0.10 | 0.54 ± 0.08 | 0.97 | 0.49 ± 0.11 | 0.48 ± 0.07 | 0.84 | 0.17 ± 0.04 | 0.17 ± 0.04 | 0.97 |
| **Lobule IV** | | | | | | | | | |
| Bilateral lobule IV | 3.73 ± 0.45 | 3.52 ± 0.26 | **0.02** | 4.17 ± 0.53 | 3.93 ± 0.35 | **0.03** | 0.59 ± 0.13 | 0.54 ± 0.09 | 0.12 |
| Right lobule IV | 1.81 ± 0.24 | 1.73 ± 0.17 | 0.14 | 2.01 ± 0.29 | 1.92 ± 0.22 | 0.17 | 0.3 ± 0.07 | 0.27 ± 0.05 | 0.20 |
| Left lobule IV | 1.91 ± 0.26 | 1.78 ± 0.17 | **0.02** | 2.16 ± 0.33 | 2.00 ± 0.21 | **0.03** | 0.29 ± 0.06 | 0.26 ± 0.05 | 0.10 |
| **Lobule V** | | | | | | | | | |
| Bilateral lobule V | 6.50 ± 0.67 | 6.28 ± 0.61 | 0.15 | 7.17 ± 0.72 | 6.92 ± 0.66 | 0.13 | 1.11 ± 0.23 | 1.05 ± 0.22 | 0.21 |
| Right lobule V | 3.20 ± 0.40 | 3.09 ± 0.33 | 0.21 | 3.45 ± 0.44 | 3.32 ± 0.36 | 0.21 | 0.60 ± 0.13 | 0.57 ± 0.12 | 0.24 |
| Left lobule V | 3.30 ± 0.36 | 3.19 ± 0.37 | 0.20 | 3.72 ± 0.38 | 3.59 ± 0.42 | 0.18 | 0.50 ± 0.11 | 0.47 ± 0.10 | 0.23 |
| **Lobule VI** | | | | | | | | | |
| Bilateral lobule VI | 14.58 ± 1.25 | 14.70 ± 1.50 | 0.69 | 16.97 ± 1.47 | 17.16 ± 1.61 | 0.58 | 1.81 ± 0.29 | 1.70 ± 0.23 | 0.09 |
| Right lobule VI | 7.34 ± 0.69 | 7.37 ± 0.81 | 0.89 | 8.45 ± 0.80 | 8.51 ± 0.86 | 0.73 | 0.98 ± 0.16 | 0.92 ± 0.14 | 0.08 |
| Left lobule VI | 7.23 ± 0.66 | 7.33 ± 0.78 | 0.55 | 8.52 ± 0.80 | 8.64 ± 0.87 | 0.50 | 0.82 ± 0.15 | 0.78 ± 0.11 | 0.23 |
| **Lobule crus I** | | | | | | | | | |
| Bilateral lobule crus I | 21.03 ± 2.18 | 20.14 ± 1.64 | 0.06 | 23.35 ± 2.37 | 22.43 ± 1.73 | 0.07 | 3.44 ± 0.50 | 3.16 ± 0.37 | **0.01** |
| Right lobule crus I | 10.61 ± 1.16 | 10.15 ± 0.82 | 0.07 | 11.68 ± 1.27 | 11.22 ± 0.84 | 0.09 | 1.81 ± 0.28 | 1.66 ± 0.22 | **0.01** |
| Left lobule crus I | 10.42 ± 1.10 | 9.99 ± 0.90 | 0.08 | 11.67 ± 1.19 | 11.21 ± 0.97 | 0.08 | 1.63 ± 0.26 | 1.50 ± 0.20 | **0.03** |
| **Lobule crus II** | | | | | | | | | |
| Bilateral lobule crus II | 13.07 ± 1.43 | 13.38 ± 1.25 | 0.33 | 15.02 ± 1.58 | 15.25 ± 1.45 | 0.52 | 1.76 ± 0.3 | 1.84 ± 0.26 | 0.24 |
| Right lobule crus II | 6.53 ± 0.81 | 6.81 ± 0.82 | 0.14 | 7.49 ± 0.92 | 7.74 ± 0.95 | 0.26 | 0.88 ± 0.17 | 0.95 ± 0.15 | 0.10 |
| Left lobule crus II | 6.54 ± 0.79 | 6.57 ± 0.72 | 0.85 | 7.52 ± 0.85 | 7.51 ± 0.81 | 0.94 | 0.87 ± 0.17 | 0.89 ± 0.17 | 0.70 |
| **Lobule VIIB** | | | | | | | | | |
| Bilateral lobule VIIB | 6.84 ± 0.72 | 7.28 ± 0.65 | **0.008** | 7.99 ± 0.81 | 8.42 ± 0.75 | **0.02** | 0.82 ± 0.15 | 0.91 ± 0.12 | **0.01** |
| Right lobule VIIB | 3.49 ± 0.46 | 3.70 ± 0.36 | **0.04** | 4.11 ± 0.53 | 4.30 ± 0.43 | 0.11 | 0.40 ± 0.09 | 0.45 ± 0.05 | **0.009** |
| Left lobule VIIB | 3.34 ± 0.38 | 3.57 ± 0.39 | **0.01** | 3.87 ± 0.42 | 4.12 ± 0.45 | **0.01** | 0.42 ± 0.09 | 0.46 ± 0.08 | 0.12 |
| **Lobule VIIIA** | | | | | | | | | |
| Bilateral lobule VIIIA | 8.66 ± 0.87 | 9.04 ± 0.98 | 0.07 | 10.00 ± 1.01 | 10.33 ± 1.14 | 0.17 | 1.14 ± 0.19 | 1.22 ± 0.20 | 0.08 |
| Right lobule VIIIA | 4.32 ± 0.49 | 4.52 ± 0.50 | 0.08 | 4.97 ± 0.57 | 5.14 ± 0.58 | 0.22 | 0.58 ± 0.11 | 0.63 ± 0.12 | **0.04** |
| Left lobule VIIIA | 4.34 ± 0.56 | 4.51 ± 0.65 | 0.21 | 5.02 ± 0.65 | 5.19 ± 0.75 | 0.28 | 0.56 ± 0.10 | 0.58 ± 0.11 | 0.36 |
| **Lobule VIIIB** | | | | | | | | | |
| Bilateral lobule VIIIB | 6.11 ± 0.90 | 6.13 ± 0.78 | 0.90 | 7.01 ± 1.04 | 6.99 ± 0.92 | 0.94 | 0.84 ± 0.18 | 0.84 ± 0.16 | 0.86 |
| Right lobule VIIIB | 3.10 ± 0.54 | 3.02 ± 0.43 | 0.51 | 3.56 ± 0.59 | 3.45 ± 0.51 | 0.41 | 0.42 ± 0.13 | 0.40 ± 0.08 | 0.71 |
| Left lobule VIIIB | 3.01 ± 0.47 | 3.11 ± 0.41 | 0.34 | 3.44 ± 0.56 | 3.53 ± 0.47 | 0.46 | 0.42 ± 0.08 | 0.43 ± 0.09 | 0.38 |
| **Lobule IX** | | | | | | | | | |
| Bilateral lobule IX | 5.40 ± 0.72 | 5.67 ± 0.93 | 0.13 | 5.74 ± 0.75 | 6.04 ± 1.05 | 0.12 | 1.08 ± 0.24 | 1.10 ± 0.24 | 0.71 |
| Right lobule IX | 2.75 ± 0.36 | 2.86 ± 0.44 | 0.21 | 2.96 ± 0.38 | 3.08 ± 0.51 | 0.20 | 0.52 ± 0.13 | 0.52 ± 0.12 | 0.89 |
| Left lobule IX | 2.65 ± 0.37 | 2.81 ± 0.50 | 0.09 | 2.77 ± 0.38 | 2.95 ± 0.54 | 0.08 | 0.56 ± 0.12 | 0.58 ± 0.13 | 0.55 |
| **Lobule X** | | | | | | | | | |
| Bilateral lobule X | 0.98 ± 0.11 | 0.98 ± 0.11 | 0.98 | 1.19 ± 0.14 | 1.17 ± 0.16 | 0.43 | 0.08 ± 0.03 | 0.09 ± 0.04 | 0.08 |
| Right lobule X | 0.49 ± 0.05 | 0.49 ± 0.06 | 0.94 | 0.59 ± 0.07 | 0.58 ± 0.09 | 0.27 | 0.04 ± 0.02 | 0.06 ± 0.02 | **0.04** |
| Left lobule X | 0.48 ± 0.05 | 0.48 ± 0.05 | 0.91 | 0.59 ± 0.07 | 0.59 ± 0.08 | 0.68 | 0.03 ± 0.01 | 0.03 ± 0.02 | 0.29 |
| * Global = both grey and white matter. | | | | | | | | | |
