# Supplementary material for: Cerebellar volume alterations are associated with cognitive dysfunction and fatigue in patients with systemic lupus erythematosus
Source: BMC Rheumatol. 2026 Jul 2;10:56. doi: 10.1186/s41927-026-00671-7 (PMC13335149; doi:10.1186/s41927-026-00671-7)
Supplement: Supplementary file 5 — Supplementary Material 5 [file 41927_2026_671_MOESM5_ESM.docx]

**Supplementary table 5**: Partial correlations between cerebellar volumes and continuous clinical scores.

| **Region-of-interest** | **Global* cerebellar volume as a percentage of total cerebellar volume** | | **Region-of-interest** | **Global* cerebellar volume as a percentage of total cerebellar volume** | |
| --- | --- | --- | --- | --- | --- |
|  | **Partial correlation coefficient (r)** | **p-value** |  | **Partial correlation coefficient (r)** | **p-value** |
| **Composite memory** | | | **Cognitive flexibility** | | |
| Number | 68 |  | Number | 68 |  |
| Bilateral lobule IV | -0.20 | 0.09 | Bilateral lobule IV | -0.11 | 0.34 |
| Left lobule IV | -0.21 | 0.08 | Left lobule IV | -0.08 | 0.50 |
| Bilateral lobule VIIB | 0.18 | 0.13 | Bilateral lobule VIIB | 0.28 | **0.02** |
| Right lobule VIIB | 0.18 | 0.12 | Right lobule VIIB | 0.31 | **0.008** |
| Left lobule VIIB | 0.12 | 0.33 | Left lobule VIIB | 0.15 | 0.21 |
| **Psychomotor speed** | | | **Pain VAS score** | | |
| Number | 68 |  | Number | 72 |  |
| Bilateral lobule IV | -0.08 | 0.52 | Bilateral lobule IV | 0.01 | 0.88 |
| Left lobule IV | -0.09 | 0.43 | Left lobule IV | -0.007 | 0.94 |
| Bilateral lobule VIIB | 0.18 | 0.13 | Bilateral lobule VIIB | -0.08 | 0.47 |
| Right lobule VIIB | 0.22 | 0.07 | Right lobule VIIB | -0.08 | 0.50 |
| Left lobule VIIB | 0.08 | 0.51 | Left lobule VIIB | -0.06 | 0.57 |
| **Reaction time** | | | **Fatigue** **VAS score** | | |
| Number | 68 |  | Number | 72 |  |
| Bilateral lobule IV | -0.24 | 0.05 | Bilateral lobule IV | 0.03 | 0.76 |
| Left lobule IV | -0.17 | 0.17 | Left lobule IV | 0.03 | 0.74 |
| Bilateral lobule VIIB | 0.20 | 0.09 | Bilateral lobule VIIB | -0.23 | **0.04** |
| Right lobule VIIB | 0.15 | 0.20 | Right lobule VIIB | -0.26 | **0.02** |
| Left lobule VIIB | 0.20 | 0.10 | Left lobule VIIB | -0.13 | 0.26 |
| **Complex attention** | | | **FSS score** | | |
| Number | 67 |  | Number | 71 |  |
| Bilateral lobule IV | -0.14 | 0.26 | Bilateral lobule IV | 0.07 | 0.55 |
| Left lobule IV | -0.07 | 0.53 | Left lobule IV | 0.10 | 0.39 |
| Bilateral lobule VIIB | 0.26 | **0.03** | Bilateral lobule VIIB | -0.15 | 0.20 |
| Right lobule VIIB | 0.31 | **0.01** | Right lobule VIIB | -0.15 | 0.19 |
| Left lobule VIIB | 0.12 | 0.32 | Left lobule VIIB | -0.09 | 0.42 |
| * Global = both grey and white matter. | | | | | |
