# Supplementary material for: Cerebellar volume alterations are associated with cognitive dysfunction and fatigue in patients with systemic lupus erythematosus
Source: BMC Rheumatol. 2026 Jul 2;10:56. doi: 10.1186/s41927-026-00671-7 (PMC13335149; doi:10.1186/s41927-026-00671-7)
Supplement: Supplementary file 6 — Supplementary Material 6 [file 41927_2026_671_MOESM6_ESM.docx]

**Supplementary table 6**: Difference in cerebellar volumes between clinical groups in SLE patients using ANCOVA.

| **Region-of-interest** | **Cerebellar global* volume as a percentage of total cerebellar volume** | | |
| --- | --- | --- | --- |
|  | **Estimated means ± SE** | | **p-value** |
| **Cognitive dysfunction in one domain** | **No cognitive dysfunction** | **Cognitive dysfunction** |  |
| Number | 32 | 36 |  |
| Bilateral lobule IV | 3.72 ± 0.08 | 3.75 ± 0.07 | 0.82 |
| Left lobule IV | 1.92 ± 0.04 | 1.92 ± 0.04 | 0.97 |
| Bilateral lobule VIIB | 6.99 ± 0.13 | 6.73 ± 0.12 | 0.14 |
| Right lobule VIIB | 3.60 ± 0.08 | 3.42 ± 0.07 | 0.12 |
| Left lobule VIIB | 3.39 ± 0.06 | 3.30 ± 0.06 | 0.36 |
| **Cognitive dysfunction in two or more domains** | **No cognitive dysfunction** | **Cognitive dysfunction** |  |
| Number | 48 | 20 |  |
| Bilateral lobule IV | 3.73 ± 0.06 | 3.74 ± 0.1 | 0.95 |
| Left lobule IV | 1.93 ± 0.03 | 1.89 ± 0.05 | 0.61 |
| Bilateral lobule VIIB | 6.91 ± 0.10 | 6.70 ± 0.16 | 0.29 |
| Right lobule VIIB | 3.55 ± 0.06 | 3.40 ± 0.10 | 0.24 |
| Left lobule VIIB | 3.36 ± 0.05 | 3.30 ± 0.08 | 0.54 |
| **MADRS-S** | **Depression score 0-6** | **Depression score > 6** |  |
| Number | 22 | 50 |  |
| Bilateral lobule IV | 3.65 ± 0.09 | 3.76 ± 0.06 | 0.32 |
| Left lobule IV | 1.85 ± 0.05 | 1.94 ± 0.03 | 0.16 |
| Bilateral lobule VIIB | 6.99 ± 0.15 | 6.77 ± 0.10 | 0.25 |
| Right lobule VIIB | 3.61 ± 0.09 | 3.44 ± 0.06 | 0.16 |
| Left lobule VIIB | 3.37 ± 0.08 | 3.33 ± 0.05 | 0.64 |
| * Global = both grey and white matter. | | | |
